# Using Salivary MMP-9 to Successfully Quantify Periodontal Inflammation during Orthodontic Treatment

**DOI:** 10.3390/jcm10030379

**Published:** 2021-01-20

**Authors:** Ionut Luchian, Mihaela Moscalu, Ancuta Goriuc, Ludovica Nucci, Monica Tatarciuc, Ioana Martu, Mihai Covasa

**Affiliations:** 1 Department of Periodontology, Grigore T. Popa University of Medicine and Pharmacy, 700115 Iasi, Romania; ionut.luchian@umfiasi.ro; 2 Department of Preventive Medicine and Interdisciplinarity, Grigore T. Popa University of Medicine and Pharmacy, 700115 Iasi, Romania; 3 Department of Biochemistry, Grigore T. Popa University of Medicine and Pharmacy, 700115 Iasi, Romania; 4 Multidisciplinary Department of Medical-Surgical and Dental Specialties, University of Campania Luigi Vanvitelli, 81100 Naples, Italy; ludovica.nucci@unicampania.it; 5 Department of Dental Technology, Grigore T. Popa University of Medicine and Pharmacy, 700115 Iasi, Romania; monica.tatarciuc@umfiasi.ro (M.T.); ioana.martu@umfiasi.ro (I.M.); 6 Department of Health and Human Development, Stefan cel Mare University of Suceava, 720229 Suceava, Romania; mcovasa@westernu.edu; 7 Department of Basic Medical Sciences, College of Osteopathic Medicine, Western University of Health Sciences, Pomona, CA 91766, USA

**Keywords:** periodontitis, saliva, matrix metalloproteinase

## Abstract

Periodontitis is one of the most common immune-mediated inflammatory conditions resulting in progressive destruction of periodontium. Metalloproteinase-9 (MMP-9), an enzyme that is involved in the degradation of gelatin and collagen and present in the gingival crevicular fluid, is markedly increased in periodontitis. The aim of the study is to evaluate the effects of periodontal treatment either alone or in combination with orthodontic treatment on MMP-9 levels. In this study, 60 individuals were subjected to periodontal treatment (PD) or periodontal treatment combined with orthodontic treatment (POD). Both periodontal and periodontal plus orthodontic treatments significantly improved clinical parameters and lowered MMP-9 levels compared to control group. However, the combination of periodontal with orthodontic treatment further improved clinical parameters and enhanced the lowering effect on MMP-9 levels compared to periodontal or control groups alone. Finally, the degree of malocclusion significantly affected the effect of the treatment on MPP-9 levels with PD treatment having the most pronounced effect. We concluded that salivary MMP-9 can serve to accurately predict the level of inflammation in affected periodontal tissues during orthodontic treatment that is also associated with the type of malocclusion, making it a viable diagnosis tool in monitoring the progression of the periodontium during orthodontic treatment.

## 1. Introduction

Periodontitis represents one of the most prevalent chronic inflammatory oral diseases. It is characterized by an inflammatory destruction of periodontal attachment complex leading to irreversible loss of bone and tooth-supporting tissue [1,2]. Among the components of periodontium lost is type I collagen, found primarily in periodontal ligament and alveolar organic matrix [3,4]. The destruction of periodontium is mediated by the plasminogen-dependent, phagocytic, osteoclastic, and matrix metalloproteinase (MMP) pathways [5,6]. The MMPs, a family of zinc and calcium-dependent proteolytic enzymes, which are secreted by immune cells in response to inflammatory stimuli, are considered the most important in mediating the degradation of the extracellular matrix [7,8] and have been recognized as important biomarkers in the early detection of several diseases [9].

Normally, these enzymes are tightly regulated and play a critical role in bone morphogenesis and tissue repair [10,11]. However, in pathological conditions such as periodontitis, these enzymes are involved in the destruction of extracellular matrix components such as collagen, elastin, fibronectin, laminin, and entactin [12,13]. This results in oral pathological processes such as the destruction of the periodontal tissue, tumor invasion, and dysfunctions of the temporomandibular joint (TMJ) [14,15]. Among these several groups of proteinases, the zinc-metalloproteinases such as matrix metallopeptidases 9 (MMP-9) contain a Zn ion in the catalytic domain. Moreover, MMP-2 and MMP-9 may have a binding domain for gelatin, inserted between the catalytic and the active domain, and this is the reason of why MMP-9 is also called gelatinase B [9]. MMP-9 or gelatinase B is primarily found in saliva and gingival crevicular fluid; it is present in dental tissues with numerous active forms, weighing 82–132 kD, and is involved in inflammation, wound healing, and tumor growth [16]. The involvement of MMP-9 both in periodontal disease and in the orthodontic periodontal reshaping has already been demonstrated by several studies [17,18,19,20]. Both in vivo and in vitro evidence show that orthodontic dental movement causes mechanical stress [21,22], which, in turn, generates biochemical and structural responses in a diversity of cell types [18,23,24]. As such, the early stage of orthodontic dental movements involves an acute inflammatory response featuring local tissular ischemia, periodontal vasodilatation, and the migration of leucocytes through the capillaries of the periodontal ligament [25]. Elevated MMPs have been associated with increased inflammation and loss of tooth-supporting tissue present in periodontal disease, while periodontal treatment decreases inflammation and lowers MMP-9 levels [23,26,27,28]. To determine whether MMP-9 levels can reliably predict the chronic inflammatory oral diseases, in this study, we examine the effects of periodontal treatment on salivary MMP-9 levels in patients with stabilized pre-existing periodontal history, where treatment is done either without or coupled with orthodontic treatment.

## 2. Materials and Methods

### 2.1. Subjects

The study was performed under the Institutional Review Board protocol no. 5329/2018 approved by the ethics committee of the Grigore T. Popa University of Medicine and Pharmacy, and signed informed consent was obtained from each participant in the study. The sample population included in the current research consists of consecutive patients that were selected in a 12-month interval. Sixty individuals of which 32 males and 28 females in good general health from 21 to 38-year-old were enrolled in the study. The following inclusion criteria were used: a minimum of 20 teeth in functional dentition, moderate or severe periodontitis and without periodontal treatment at the time of enrolment. Periodontal exam included the recording of periodontal pocket depths (PPD) from six sites of each tooth. The six probing sites were distributed as follows: three sites on the buccal surfaces (mesial, central, and distal) and three sites on the lingual surfaces of the teeth (mesial, central, and distal). Bleeding on probing was recorded and the sulcus bleeding index (SBI) was determined. A complete periodontal probing was performed using an electronic probe (PaOn^®,^ Orange Dental, Biberach a. d. Riss, Baden-Würtemberg State, Germany), and data were transferred using additional software. The following exclusion criteria were applied: smoking, heavy drinkers, immunocompromised, use of anti-inflammatory drugs or other medication affecting the periodontium, use of antibiotics and steroids, and a history of systemic and infectious diseases.

The sixty patients included in the study were randomly divided in three groups as follows: group 1, a control group that included 16 (7 men, 9 women) subjects without periodontal disease and/or clinical gingival modifications; group 2, which included 22 subjects (10 men, 12 women) with periodontal disease (chronic periodontitis localized in minimum 3 teeth) who received periodontal treatment (PD); and group 3, which included 22 (11 men, 11 women) subjects with periodontal disease (chronic periodontitis localized in minimum 3 teeth), who received both periodontal and orthodontic treatment (POD). All individuals received periodontal examination and were diagnosed based on the clinical criteria established by the American Academy of Periodontology [29]. All patients underwent treatment including oral hygiene instructions. Periodontal treatment was identical for both PD and POD groups and consisted of supra- and subgingival scaling and root planning over a maximum 4-week period. The subgingival scaling was performed using the same ultrasonic device (Acteon Satelec^®^, Mérignac, Gironde, France), and the same type of subgingival inserts while for root planning area specific curettes (Hu-Friedy^®^, Chicago, IL, USA) were used. Therapeutic treatments were performed every 8 weeks starting 10 days after study commencement. For orthodontic treatment, we used metallic fixed appliances that were bonded after the periodontal status stabilized. No dropouts occurred prior or during the treatment.

### 2.2. Saliva Sample Collection and Analysis

Saliva was collected at two time points from patients included in PD and POD groups as follows: for the PD group, one initial baseline collection and a second collection 6 months following completion of periodontal treatment; for the POD group, one initial baseline collection and a second collection 6 months after completion of periodontal treatment and stabilization of the orthodontic treatment. The timing for the second saliva collection in the POD group was delayed compared to the PD group to allow for an evaluation of the effects of the orthodontic treatment on the inflammatory markers level once the treatment has stabilized. One baseline saliva collection was performed in the control group at the beginning of the study. All samples were collected by one investigator to ensure consistency in the protocol. Saliva was collected without stimulating its secretion from the salivary glands (i.e., paraffin gum or citric acid) in order to avoid any interference of the stimulating agent on marker release. Collection was carried out using Eppendorf tubes placed on ice; particular attention was given to contamination with blood, since MPP is also present in blood and its levels were shown to be different (i.e., higher) in blood compared to those present in the salivary fluid. Samples suspected of blood contamination were discarded. Samples were stored at −20 °C pending analysis. The levels of MMP-9 in collected saliva samples were measured via ELISA immunoassay following the manufacturer’s instructions (R&D Systems Inc., Minneapolis, MN, USA) [19].

### 2.3. Statistical Analyses

Statistical analyses were performed using SPSS 24.0 for Windows (IBM Corporation, North Castle Drive, Armonk, NY, USA). The Kruskal–Wallis test was applied to determine the differences in MMP concentrations between groups of patients according to treatment and clinical parameters. The Newman–Keuls post hoc test was also applied for the pair analysis of two groups of patients. MMP-9 values were reported as mean values and standard deviation. The evolution of MMP-9 values was also presented in %, with the proportion being represented by the decrease of the MMP-9 value related to the value registered before the treatment. The univariate correlational analysis was performed based on the Spearman rank order correlations test. To better highlight the effect of the treatment and the degree of malocclusion, the graphs were generated using STATA 16.1 (StataCorp LLC., College Station, TX, USA). A *p*-value of less than 0.05 was considered statistically significant.

## 3. Results

### 3.1. Clinical Parameters

Analysis showed a significant difference before treatment between PD and control groups for PPD (4.18 ± 0.21 vs. 1.65 ± 0.14, *p* < 0.0001) and SBI (2.9 ± 0.11, vs. 0.31 ± 0.11, *p* < 0.0001) as well as between POD and control groups for the same parameters: PPD = 4.54 ± 0.17, *p* < 0.0001; SBI = 3.45 ± 0.12, *p* < 0.001. Periodontitis treatment significantly improved PPD (3.23 ± 0.19, *p* < 0.01) and SBI (2.04 ± 0.12, *p* < 0.01) while periodontitis combined with orthodontic treatment had a greater effect compared to the control group and the PD group’s treatment for both clinical parameters, i.e., PPD = 2.4 ± 0.1, SBI = 2.04 ± 0.12, *p* < 0.0001 for both.

### 3.2. Effects of Periodontal and Combined Periodontal with Orthodontic Treatment on MMP-9 Levels

**Figure 1:**
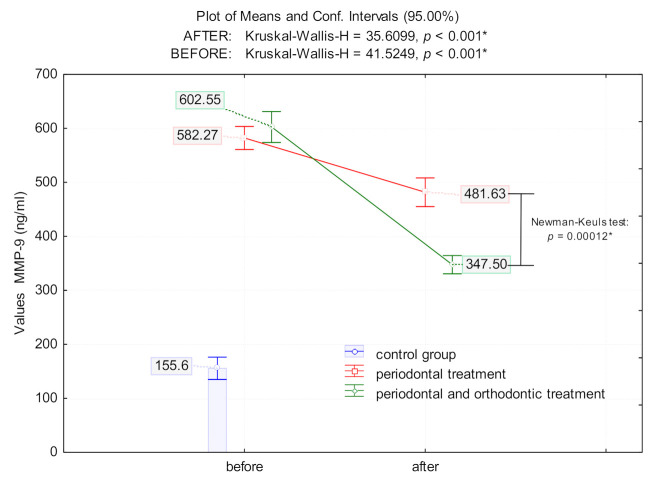
The means value of MMP-9 [ng/mL] in patients with periodontal disease prior and after periodontal treatment (PD) and periodontal and orthodontic treatment (POD) treatment; (*) indicates that marked effects are significant at *p* < 0.05.

Patients with untreated periodontal disease had significantly higher levels of salivary MMP-9 compared to controls (control group: 155.6 ± 38.63 ng/mL; PD: 582.27 ± 48.2 ng/mL; POD group: 602.55 ± 64.55 ng/mL; *p* < 0.0001 for both, Figure 1). However, following intervention, periodontal treatment alone lowered MMP-9 significantly compared to the levels before treatment (17.3% reduction; *p* = 0.0046). A combination of periodontal with orthodontic treatment drastically decreased MMP-9 levels by 42.3% compared to pre-treatment levels (*p* < 0.0001). Although MMP-9 levels dropped significantly after treatment in both PD and POD groups, there was a significant difference (*p* = 0.00012) in MMP-9 levels following each of the two treatments, with the treatment for the POD group having the most significant effect in lowering MMP-9 levels compared to the PD and control groups (*p* = 0.0005) (Figure 1).

### 3.3. The Effect of Malocclusion on MMP-9 Levels

**Figure 2:**
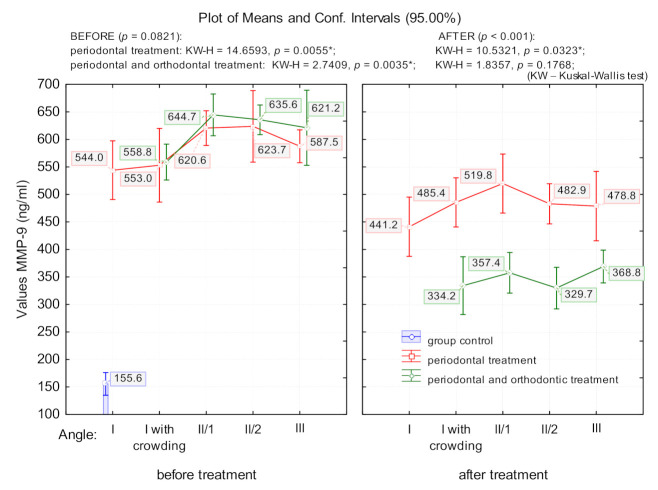
The means value of MMP-9 in patients with periodontal disease, in relation to angle class; (*) indicates that marked effects are significant at *p* < 0.05.

The degree of malocclusion significantly affected salivary MMP-9 levels. Because of this, prior to treatment, patients with periodontal disease had MMP-9 values that differed significantly depending on the angle class (PD group: *p* = 0.005; POD group: *p* = 0.003); this effect was more pronounced in patients with angle class II/1 and II/2 (Figure 2). Following the PD group’s treatment, MMP-9 decreased significantly compared as a function of the angle class (*p* = 0.03). The treatment in the POD group significantly lowered MMP-9 levels compared to those of the PD and control groups (*p* < 0.0001); however, there were no significant differences between the angle classes (*p* = 0.176) (Figure 2).

### 3.4. Correlation Between Clinical Parameters and MMP-9 Levels

Before treatment, the Spearman rank analyses showed a significant positive association between the probing pocket depth (PPD) and MMP-9 levels in POD patients (*r* = 0.47, *p* = 0.03) with no difference in the PD group (*r* = 0.033, *p* = 0.88). Following intervention, MMP-9 levels were significantly correlated with PPD in the PD group (*r* = 0.33, *p* = 0.029) but not in the POD group (*r* = 0.678, *p* = 0.764). Similarly, there was a significant correlation between SBI and MMP-9 levels before treatment in the POD group (*r* = 0.47, *p* = 0.032) but not the PD group (*r* = 0.08, *p* = 0.71). After treatment in the PD group, MMP-9 levels in the PD group were significantly correlated with SBI (*r* = 0.46, *p* = 0.034) while the treatment in POD group abrogated the correlation between SBI and MMP-9.

## 4. Discussion

The results of this study show that both periodontal and the combination of periodontal and orthodontic treatments were effective in significantly lowering MMP-9 levels compared to patients who did not receive the treatment. However, it was the combination of periodontal with orthodontic treatment that had the greatest effect on MMP-9 levels compared to periodontal or control groups alone. Furthermore, while the treatment in both the PD and POD groups significantly improved clinical parameters, the POD group treatment had the greatest effect. This improvement of clinical parameters following intervention in the PD and POD groups was positively associated with MMP-9 levels. Finally, the degree of malocclusion significantly affected the effect of the treatment on MPP-9 levels with PD treatment having the most pronounced effect.

Periodontitis is one of the most prevalent inflammatory pathology affecting nearly half of people over 30 years old [3]. It is characterized by an immune inflammatory process ultimately leading to the destruction of periodontal attachment and supporting tissue and bone resorption. Several periodontal bacteria together with microbial proteases such as metalloproteases including host-derived MMPs, all participate in the process leading to progression of periodontitis, tissue, and ligament degradation. Among them are *Aggregatibacter actinomycetemcomitans*, *Porphyromonas gingivalis*, *Treponema denticola*, and *Tannerella forsythia*; together with the host’s genetics and other environmental factors, these bacteria are active contributors in the infections and inflammatory processes that result in the production of metalloproteases, including host MMPs [16,30,31]. MMP-9 is among the best studied proteinases when it comes to its role in periodontitis and its activation in infections.

In the present study, salivary MMP-9 levels were associated significantly with periodontal and orthodontic treatment and differentiated from the control group. Therefore, MMP-9 can be used with high fidelity not only as a marker of periodontal inflammation but also as a tool for assessing the effectiveness of the nonsurgical periodontal and orthodontic therapy. The increased MMPs, whether in saliva or serum samples in periodontitis, were well documented. For example, salivary as well as circulating MMP-8 and MMP-9 levels were significantly elevated in periodontitis patients [32] and reduced after periodontal treatment [19,33,34].

The use of MMPs as salivary markers for local or systemic pathologies such as CVDs, diabetes, dyslipidemia has been of interest for sometimes, but not without challenges, given their vulnerability against local inhibitors in periodontitis condition that can limit their use in systemic diseases [35]. In addition to the periodontitis group that was associated with increased MMP-9, patients who received both periodontal as well as orthodontic treatment displayed an augmentation in the inhibition of MMP-9 levels, compared with either treatment alone. To our knowledge, this is the first report demonstrating that combination of periodontal and orthodontic treatment is associated with a reduction in MMP-9 levels.

Previous work has shown increased salivary biomarkers in orthodontic treatment involving alignment with fixed appliances, which have been associated with tooth movement [36,37]. This is not surprising since there is increased osteoclast activity when orthodontic forces are applied, and MMP-9 is expressed in osteoclasts where it controls proteolysis in bone resorption. Therefore, inflammatory markers such as MMP-9 are associated with osteoclasts activity, a phenomenon present in response to orthodontic applied forces [17,38]. In vitro studies showed an increase in MMP-9 following application of orthodontic forces, an effect that is dependent on the degree of tension and compression [39]. In our study, however, we measured MMP-9 levels after orthodontic treatment was completed and patients stabilized. This resulted in a further decrease in the MMP-9 levels in patients who received orthodontic treatment after periodontitis was improved or resolved. These results are also in line with findings demonstrating that MMP-9 levels decreased significantly in patients with periodontitis with orthodontic restorations [40] and that MMP-9 levels oscillated during application of orthodontic forces and decreased as early as 24h after appliance activation [41].

Another important aspect of the study is highlighted by the findings that the MMP-9 values following the combined treatment dropped significantly more for all angle classes, with no significant differences between groups, whereas in the case of periodontal treatment MMP-9 values remain elevated in the patients who had angle classes II and III malocclusions. A comparative analysis of MMP-9 values in relation to the type of treatment and to the angle class shows that after combined periodontal and orthodontic treatment, the values of MMP-9 lowered significantly more, despite the fact that they were significantly higher before treatment.

Therefore, although at the start of the treatment patients with periodontal problems who were about to begin periodontal treatment combined with orthodontic treatment had higher values of MMP-9 (although not significant), these values dropped significantly more compared to those of patients who had only periodontal treatment. The persistence of high values of MMP-9 in patients who received only periodontal treatment, particularly in the case of those with angle classes II/2 and II/1 malocclusions, as well as those with angle class III malocclusions, is convincing and clearly demonstrates that orthodontic treatment combined with periodontal treatment significantly reduces inflammation in the affected periodontal tissues.

A complex analysis to assess changes in MMP-9 values in chronic inflammatory oral diseases could be performed using both MMP-9 levels and the type of treatment, in a clustered form. This method could lead to a significant increase in the prediction of the evolution of periodontal disease [42].

Existing literature shows that both the saliva tests and the tests performed on crevicular fluid provide valuable diagnosis information concerning the stage of the inflammation in periodontal disease. Several authors have shown that metalloproteinase-8 (MMP-8), an enzyme responsible for tissue destruction, was positively associated with periodontal disease [43,44,45]. Because of this, an immunochromatography assay was developed and is commercially available for assessing MMP-8 in crevicular fluid. This test can be carried out in clinical practice and has the same accuracy as a laboratory test. This facilitates testing of this particular metalloproteinase and opens new perspectives in terms of the predictability of the chosen treatment plan [46]. Currently, no similar test/assay exists for determining MMP-9 in saliva or in the crevicular fluid.

## 5. Conclusions

In conclusion, our results point to salivary MMP-9 as a strong candidate for quantifying inflammation in affected periodontal tissues during orthodontic treatment. It further indicates that MMP-9 can be used to accurately predict the level of inflammation associated with the type of malocclusion, which makes it a real and viable diagnosis instrument in monitoring the evolution of the periodontium during orthodontic treatment. Larger scale studies conducted on patients with various degrees of periodontitis and orthodontic treatments are needed to further establish the use of salivary MMP-9 as a predictor of inflammation following orthodontic treatment.

## References

[1] Könönen E., Gursoy M., Gursoy U.K. (2019). Periodontitis: A Multifaceted Disease of Tooth-Supporting Tissues. J. Clin. Med..

[2] Kim J.Y., Kim H.N. (2020). Changes in Inflammatory Cytokines in Saliva after Non-Surgical Periodontal Therapy: A Systematic Review and Meta-Analysis. Int. J. Environ. Res. Public Health.

[3] Papapanou P.N., Susin C. (2017). Periodontitis epidemiology: Is periodontitis under-recognized, over-diagnosed, or both?. Periodontol. 2000.

[4] Benjamin R.M. (2010). Oral health: The silent epidemic. Public Health Rep..

[5] Uitto V.J., Overall C.M., McCulloch C. (2003). Proteolytic host cell enzymes in gingival crevice fluid. Periodontology 2000.

[6] Minervini G., Nucci L., Lanza A., Femiano F., Contaldo M., Grassia V. (2020). Temporomandibular disc displacement with reduction treated with anterior repositioning splint: A 2-year clinical and magnetic resonance imaging (MRI) follow-up. J. Biol. Regul. Homeost. Agents.

[7] Boelen G.J., Boute L., d’Hoop J., EzEldeen M., Lambrichts I., Opdenakker G. (2019). Matrix metalloproteinases and inhibitors in dentistry. Clin. Oral. Investig..

[8] Moccia S., Nucci L., Spagnuolo C., d’Apuzzo F., Piancino M.G., Minervini G. (2020). Polyphenols as Potential Agents in the Management of Temporomandibular Disorders. Appl. Sci..

[9] Laronha H., Caldeira J. (2020). Structure and Function of Human Matrix Metalloproteinases. Cells.

[10] Birkedal-Hansen H. (1993). Role of matrix metalloproteinases in human periodontal diseases. J. Periodontol..

[11] Sorsa T., Tjaderhane L., Salo T. (2004). Matrix metalloproteinases (MMPs) in oral diseases. Oral. Dis..

[12] Rathnayake N., Gustafsson A., Norhammar A., Kjellstrom B., Klinge B., Ryden L., Tervahartiala T., Sorsa T., Group P.S. (2015). Salivary Matrix Metalloproteinase-8 and -9 and Myeloperoxidase in Relation to Coronary Heart and Periodontal Diseases: A Subgroup Report from the PAROKRANK Study (Periodontitis and Its Relation to Coronary Artery Disease). PLoS ONE.

[13] Narayanan A.S., Page R.C. (1983). Connective tissues of the periodontium: A summary of current work. Coll. Relat. Res..

[14] Aiba T., Akeno N., Kawane T., Okamoto H., Horiuchi N. (1996). Matrix metalloproteinases-1 and -8 and TIMP-1 mRNA levels in normal and diseased human gingivae. Eur. J. Oral. Sci..

[15] Escalona L.A., Mastromatteo-Alberga P., Correnti M. (2016). Cytokine and metalloproteinases in gingival fluid from patients with chronic periodontitis. Investig. Clin..

[16] Sorsa T., Tjaderhane L., Konttinen Y.T., Lauhio A., Salo T., Lee H.M., Golub L.M., Brown D.L., Mantyla P. (2006). Matrix metalloproteinases: Contribution to pathogenesis, diagnosis and treatment of periodontal inflammation. Ann. Med..

[17] Grant M., Wilson J., Rock P., Chapple I. (2013). Induction of cytokines, MMP9, TIMPs, RANKL and OPG during orthodontic tooth movement. Eur. J. Orthod..

[18] Lahdentausta L.S.J., Paju S., Mantyla P., Buhlin K., Tervahartiala T., Pietiainen M., Alfthan H., Nieminen M.S., Sinisalo J., Sorsa T. (2018). Saliva and serum biomarkers in periodontitis and coronary artery disease. J. Clin. Periodontol..

[19] Marcaccini A.M., Meschiari C.A., Zuardi L.R., de Sousa T.S., Taba M., Teofilo J.M., Jacob-Ferreira A.L., Tanus-Santos J.E., Novaes A.B., Gerlach R.F. (2010). Gingival crevicular fluid levels of MMP-8, MMP-9, TIMP-2, and MPO decrease after periodontal therapy. J. Clin. Periodontol..

[20] Grassia V., D’Apuzzo F., Ferrulli V.E., Matarese G., Femiano F., Perillo L. (2014). Dento-skeletal effects of mixed palatal expansion evaluated by postero-anterior cephalometric analysis. Eur. J. Paediatr. Dent..

[21] Maspero C., Fama A., Cavagnetto D., Abate A., Farronato M. (2019). Treatment of dental dilacerations. J. Biol. Regul. Homeost. Agents.

[22] Maspero C., Abate A., Cavagnetto D., Fama A., Stabilini A., Farronato G., Farronato M. (2019). Operculectomy and spontaneous eruption of impacted second molars: A retrospective study. J. Biol. Regul. Homeost. Agents.

[23] Alikhani M., Chou M.Y., Khoo E., Alansari S., Kwal R., Elfersi T., Almansour A., Sangsuwon C., Al Jearah M., Nervina J.M. (2018). Age-dependent biologic response to orthodontic forces. Am. J. Orthod. Dentofac. Orthop..

[24] Perinetti G., D’Apuzzo F., Contardo L., Primozic J., Rupel K., Perillo L. (2015). Gingival crevicular fluid alkaline phosphate activity during the retention phase of maxillary expansion in prepubertal subjects: A split-mouth longitudinal study. Am. J. Orthod. Dentofac. Orthop..

[25] Di Domenico M., D’Apuzzo F., Feola A., Cito L., Monsurro A., Pierantoni G.M., Berrino L., De Rosa A., Polimeni A., Perillo L. (2012). Cytokines and VEGF induction in orthodontic movement in animal models. J. Biomed. Biotechnol..

[26] Balli U., Keles G.C., Cetinkaya B.O., Mercan U., Ayas B., Erdogan D. (2014). Assessment of vascular endothelial growth factor and matrix metalloproteinase-9 in the periodontium of rats treated with atorvastatin. J. Periodontol..

[27] Meschiari C.A., Marcaccini A.M., Santos Moura B.C., Zuardi L.R., Tanus-Santos J.E., Gerlach R.F. (2013). Salivary MMPs, TIMPs, and MPO levels in periodontal disease patients and controls. Clin. Chim. Acta.

[28] Goncalves P.F., Huang H., McAninley S., Alfant B., Harrison P., Aukhil I., Walker C., Shaddox L.M. (2013). Periodontal treatment reduces matrix metalloproteinase levels in localized aggressive periodontitis. J. Periodontol..

[29] Greenwell H., Committee on Research, Science and Therapy, American Academy of Periodontology (2001). Position paper: Guidelines for periodontal therapy. J. Periodontol..

[30] Rathnayake N., Gieselmann D.R., Heikkinen A.M., Tervahartiala T., Sorsa T. (2017). Salivary Diagnostics-Point-of-Care diagnostics of MMP-8 in dentistry and medicine. Diagnostics (Basel).

[31] Cobzeanu B.M., Costan V.V., Danciu M., Pasca A.S., Sulea D., Ungureanu L.B., Moscalu M., Cobzeanu M.D., Popescu E. (2017). Environmental factors involved in genesis of retromolar—oropharynx junction cancer. Environ. Eng. Manag. J..

[32] Marcaccini A.M., Novaes A.B., Meschiari C.A., Souza S.L., Palioto D.B., Sorgi C.A., Faccioli L.H., Tanus-Santos J.E., Gerlach R.F. (2009). Circulating matrix metalloproteinase-8 (MMP-8) and MMP-9 are increased in chronic periodontal disease and decrease after non-surgical periodontal therapy. Clin. Chim. Acta.

[33] Correa F.O.B., Goncalves D., Figueredo C.M.S., Gustafsson A., Orrico S.R.P. (2008). The Short-Term Effectiveness of Non-Surgical Treatment in Reducing Levels of Interleukin-1beta and Proteases in Gingival Crevicular Fluid from Patients with Type 2 Diabetes Mellitus and Chronic Periodontitis. J. Periodontol..

[34] Figueredo C.M., Areas A., Miranda L.A., Fischer R.G., Gustafsson A. (2004). The short-term effectiveness of non-surgical treatment in reducing protease activity in gingival crevicular fluid from chronic periodontitis patients. J. Clin. Periodontol..

[35] Miller C.S., Foley J.D., Bailey A.L., Campell C.L., Humphries R.L., Christodoulides N., Floriano P.N., Simmons G., Bhagwandin B., Jacobson J.W. (2010). Current developments in salivary diagnostics. Biomark Med..

[36] Kapoor P., Kharbanda O.P., Monga N., Miglani R., Kapila S. (2014). Effect of orthodontic forces on cytokine and receptor levels in gingival crevicular fluid: A systematic review. Prog. Orthod..

[37] Saloom H.F., Carpenter G.H., Cobourne M.T. (2019). A cross-sectional cohort study of gingival crevicular fluid biomarkers in normal-weight and obese subjects during orthodontic treatment with fixed appliances. Angle Orthod..

[38] Takahashi I., Onodera K., Nishimura M., Mitnai H., Sasano Y., Mitani H. (2006). Expression of genes for gelatinases and tissue inhibitors of metalloproteinases in periodontal tissues during orthodontic tooth movement. J. Mol. Histol..

[39] Kapoor P., Monga N., Kharbanda O.P., Kapila S., Miglani R., Moganty R. (2019). Effect of orthodontic forces on levels of enzymes in gingival crevicular fluid (GCF): A systematic review. Dental. Press J. Orthod..

[40] Kushlinskii N.E., Solovykh E.A., Karaoglanova T.B., Boyar U., Gershtein E.S., Troshin A.A., Maksimovskaya L.N., Yanushevich O.O. (2012). Matrix metalloproteinases and inflammatory cytokines in oral fluid of patients with chronic generalized periodontitis and various construction materials. Bull. Exp. Biol. Med..

[41] Capelli J., Kantarci A., Haffajee A., Teles R.P., Fidel R., Figueredo C.M. (2011). Matrix metalloproteinases and chemokines in the gingival crevicular fluid during orthodontic tooth movement. Eur. J. Orthod..

[42] Boiculese L.V., Dimitriu G., Moscalu M. (2009). Nearest neighbor classification with improved weighted dissimilarity measure. Proc. Rom. Acad. Ser. A.

[43] Herr A.E., Hatch A.V., Throckmorton D.J., Tran H.M., Brennan J.S., Giannobile W.V., Singh A.K. (2007). Microfluidic immunoassays as rapid saliva-based clinical diagnostics. Proc. Natl. Acad. Sci. USA.

[44] Kinane D.F., Darby I.B., Said S., Luoto H., Sorsa T., Tikanoja S., Mantyla P. (2003). Changes in gingival crevicular fluid matrix metalloproteinase-8 levels during periodontal treatment and maintenance. J. Periodontal. Res..

[45] Prescher N., Maier K., Munjal S.K., Sorsa T., Bauermeister C.D., Struck F., Netuschil L. (2007). Rapid quantitative chairside test for active MMP-8 in gingival crevicular fluid: First clinical data. Ann. N. Y. Acad. Sci..

[46] Sorsa T., Hernandez M., Leppilahti J., Munjal S., Netuschil L., Mantyla P. (2010). Detection of gingival crevicular fluid MMP-8 levels with different laboratory and chair-side methods. Oral. Dis..

